# A Rare Concomitant Oncocytic Adrenocortical Neoplasm and Hepatocellular Carcinoma over a Four-year Duration: A Case Report and Review of Literature

**DOI:** 10.1155/2019/9137120

**Published:** 2019-10-20

**Authors:** Daniyah Saleh, Wafaey Gomaa, Jaudah Al-Maghrabi

**Affiliations:** ^1^Department of Anatomic Pathology, King Faisal Specialist Hospital and Research Center, Jeddah, Saudi Arabia; ^2^Department of Pathology, Faculty of Medicine, King Abdulaziz University, Jeddah, Saudi Arabia; ^3^Department of Pathology, Faculty of Medicine, Minia University, Al Minia, Egypt

## Abstract

Oncocytic adrenocortical neoplasms (OANs) are very rare. Although most cases have benign behavior, the risk of recurrence/metastasis is variable. Based on Lin-Weiss-Bisceglia (LWB) system criteria, OANs can be classified as benign, borderline, or malignant. A concomitant development of OANs with second primary neoplasm is extremely uncommon, and is limited to very few case reports. None of these reported cases was found to be associated with hepatocellular carcinoma (HCC). In this case report, we present a 64-year-old female patient who had a progressively increasing left supra-renal mass over a three-year interval. During her regular imaging-based follow up after successful left adrenalectomy, a new suspicious solitary, hypodense liver mass was detected and removed. All necessary work-up was done and strongly support the diagnosis of two distinct primary tumors including borderline malignant potential OAN and subsequent HCC. A significant clinical and morphological characteristic of OANs make its identification valuable.

## 1. Introduction

Oncocytic neoplasms are defined as tumors rich with specific type of epithelial cells known as oncocytes. Oncocytes have abundant granular eosinophilic cytoplasm. Ultrastructurally, oncocytes possess numerous cytoplasmic mitochondria. Oncocytes can be arranged in various growth patterns such as diffuse sheet-like, alveolar, trabecular, and glandular [1]. Oncocytic neoplasms are rare neoplasms that have been described in different organs throughout the body, most frequently in kidneys, thyroid, parathyroid, or salivary glands, as well as other sites [2]. Oncocytic adrenocortical neoplasms (OANs) originate from adrenal cortex are extremely rare. They are usually nonfunctioning tumors with majority of cases found in adults with female predominance. Lin-Weiss-Bisceglia (LWB) system has been developed in 2004 as a simple robust system for assessment of OANs' malignant potential. This system proposes that the presence of at least 1 of the 3 major criteria (mitotic rate greater than 5 per 50 HPF, atypical mitoses, and venous invasion) is indicative of malignancy, while at least 1 of the 4 minor criteria (size greater than 10 cm and/or weight greater than 200 g, microscopic necrosis, capsular invasion, and sinusoidal invasion) is indicative of borderline malignant potential, and the absence of all criteria is indicative of a benign neoplasm. OANs likely carry a better prognosis compared to their nononcocytic counterpart [3]. Hepatocellular carcinoma (HCC) is the most common form of primary liver cancer. The incidence rates of HCC are slowly increasing worldwide [4]. HCC have been observed mostly (80%) in sub-Saharan Africa and in Eastern Asia population [5]. The majority of HCC arise in a cirrhotic liver as a result of particular important risk factors as chronic hepatitis B virus or hepatitis C virus infections [6]. None of the known syndromes or hereditary conditions is associated with co-occurrence of OANs and HCC. Herein, we report a case found to have two unrelated primary tumors consisting of an OAN and HCC in a four-year interval.

## 2. Case Presentation

**Figure 1:**
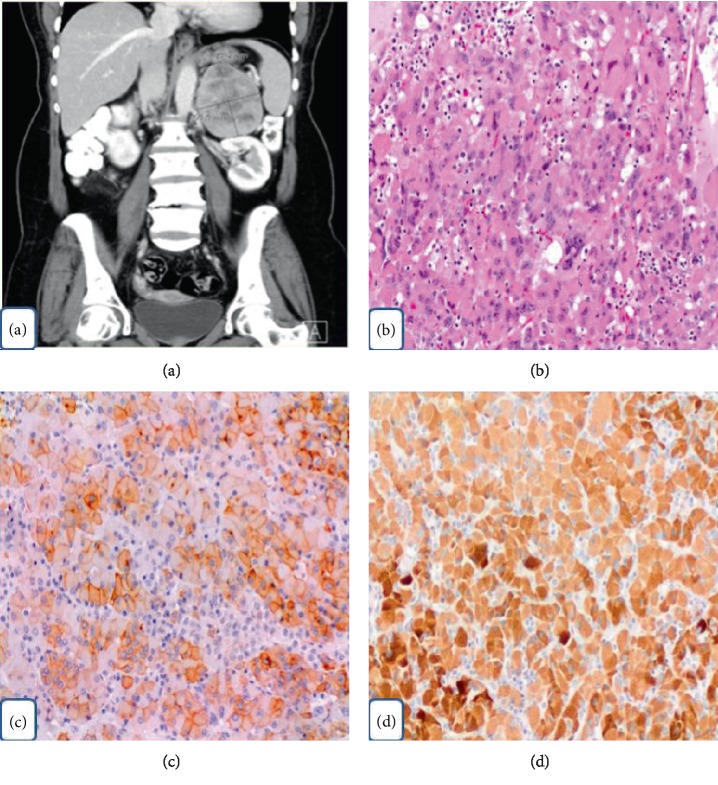
(a) CT scan of abdomen illustrates the left supra-renal mass. (b) Oncocytic adrenocortical neoplasm (H&E 100x). (c) Tumor cells expressing positive staining for synaptophysin (100x). (d) Tumor cells expressing positive staining for Melan-A (100x).

**Figure 2:**
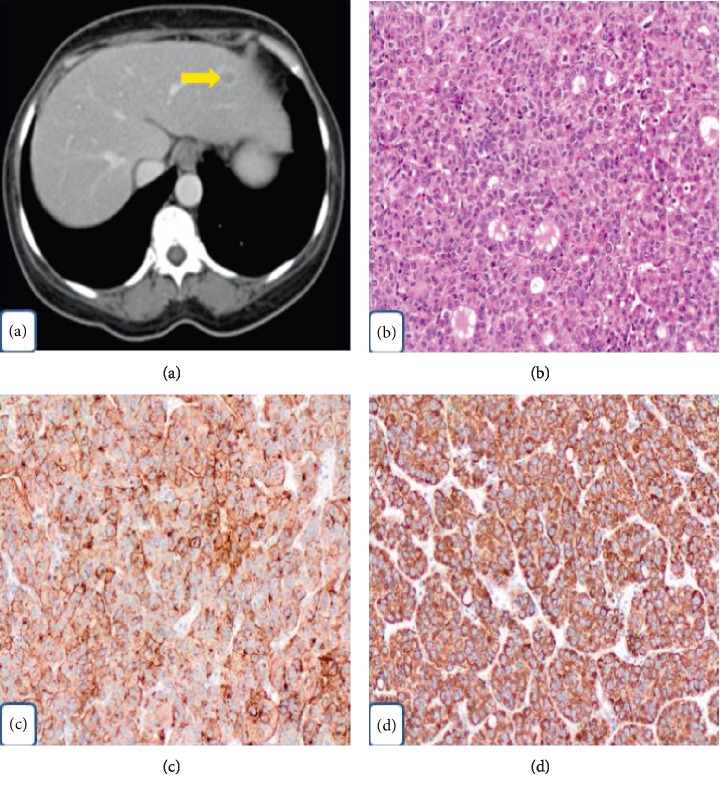
(a) CT scan of abdomen with contrast illustrates solitary hypodense liver lesion in left lobe (yellow arrow). (b) Hepatocellular carcinoma showing pseudoglandular (acinar) pattern (H&E; 100x). (c) Tumor cells expressing positive staining for Glypican-3 (100x). (d) Tumor cells expressing strong positive staining for HepPar-1 (100x).

The case represents a 64-year-old female patient known with hepatitis C virus infection, liver cirrhosis, hypertension, type 2 diabetes mellitus, and hypothyroidism on medications. In 2015, she presented to surgery clinic complaining of left flank mass associated with recurrent abdominal pain in the previous three years. By physical examination, the left abdominal mass was palpable with tenderness. The mass was progressively increasing in size from 5 cm to ~15 cm in greatest dimension in a three-year interval. Liver enzymes (AST and ALT) were high. Vital signs were within normal limits. Since the patient is hypertensive, pheochromocytoma was clinically suspected. Accordingly, urine analysis of metanephrine was done and it was within normal range. Further radiological investigations through abdominal computed tomography scan revealed a markedly enlarged heterogeneous left supra-renal mass (8.9 × 8.5 × 7.5 cm), for which, she underwent exploratory laparotomy and complete excision of the mass and sent for histopathology evaluation. The patient gave consent prior surgery. Grossly, the mass measured 10 × 7.5 × 5 cm, well-circumscribed, and encapsulated. Cut section showed a lobulated, yellow/tan to orange surface with foci of hemorrhage. No necrosis was seen. A portion of adrenal gland was found attached to the outer surface of the mass measuring 1.3 × 0.7 × 0.5 cm. Microscopic examination revealed a neoplasm composed predominantly of diffuse polygonal cells with abundant granular and eosinophilic cytoplasm. They have large nuclei and prominent nucleoli. Occasional mononuclear and binucleated giant cells are seen. Neither nether vascular invasion nor necrosis was identified. Rare mitotic figures are noted (Figure 1). The morphological differential diagnosis includes OAN and oncocytic pheochromocytoma. An expanded panel of immunohistochemical markers was performed. The tumor cells were immunoreactive to CD56, synaptophysin, calretinin, and melan-A. On the other hand, they were negative to pankeratin, S-100, chromogranin, inhibin, CK7, CK19, PAX-8, EMA, CD117, HMB-45, Glypican-3, HepPar-1, and Bcl-2. The proliferative index (Ki-67) is <1% of the tumor cells. Immunohistochemical features were more in favor with OAN. There are no convincing features of malignancy in this neoplasm; indeed, regarding LWB system, the size of the tumor considers a minor criterion in assessment of malignant potential in OANs. The morphological features and immunostaining supported the diagnosis of borderline malignant potential with <10% risk of recurrence/metastasis. Moreover, the patient was doing-well on her regular postoperative clinical and imaging-based follow-up. However, in 2019 subsequent CT scan of the abdomen showed a suspicious small, solitary, and hypodense liver lesion in segment 7 of left lobe. A laparotomy was performed and the hepatic lesion was excised. Grossly, cut section of the liver segment showed multiple well-circumscribed, whitish, and firm nodules. The largest nodule measures 2 × 1.1 × 1 cm and the smallest measures 0.4 × 0.2 × 0.2 cm. Microscopic examination exhibits neoplastic cells arranged in nodular pattern. The cells are polygonal with distinct cell membranes, abundant granular eosinophilic cytoplasm. They have high N/C ratio, round nuclei with coarse chromatin, and thickened nuclear membrane; some have prominent nucleoli. Sinusoidal vessels surrounding tumor cells are seen. The neoplastic cells are separated by fibrous septae with a background of cirrhotic liver. A panel of immunohistochemical markers was performed in order to rule out a metastatic adrenocortical neoplasm. Tumor cells were positive to glypican-3 and hepar-1 and negative to synaptophysin, melan-A, and chromogranin (Figure 2). Based on the above immunostaining profile, the diagnosis of moderately differentiated HCC was confirmed.

## 3. Discussion

According to the available literatures, many studies emphasizing the importance of recognition of OANs. The LWB system is applied for assessment of malignant potential of OANs. A malignant OANs present with any of major criteria (mitotic rate greater than 5 per 50 HPF, atypical mitoses, and venous invasion), borderline malignant potential present with at least 1 of the 4 minor criteria (size greater than 10 cm and/or weight greater than 200 g, microscopic necrosis, capsular invasion, and sinusoidal invasion), and neither of major nor minor criteria present in a benign oncocytoma. OANs have been described in adults in their fourth-sixth decades of life with Female: male ratio of 1.8 : 1. Left adrenal gland is more affected than right adrenal gland. Most OANs are nonfunctioning and usually incidentally discovered during abdominal imaging. A small percentage of hormonal secreting adrenocortical neoplasms present with various clinical symptoms such as Cushing syndrome, pheochromocytoma-like syndrome, and virilisation [3]. Most OANs are benign. The risk of recurrence/metastasis is null in benign, 3% in borderline, and 15 in malignant [3]. OANs with borderline malignant potential have a benign clinical behavior. These tumors require long-term follow-up and a thorough clinical, hormonal, and imaging evaluation [7]. In the current case, based on the characteristics microscopic features of oncocytic neoplasms are seen including large, polygonal cells with abundant granular, eosinophilic cytoplasm arranged in sheets-like pattern; oncocytic pheochromocytoma was the main differential diagnosis. However, the negative stain for chromogranin and positive stain for CD56 and synaptophysin ruled out pheochromocytoma. OANs are typically immunoreactive for vimentin, synaptophysin, melan A, and inhibin-*α*. Variable positivity is seen for pancytokeratin anti-bodies CK8, CK18, and CD10. Immunostaining for CK20, chromogranin, S-100 protein, HMB-45, and EMA is usually negative. The current case shows a similar immunoprofile pattern. The OANs' immunohistochemical profile is identical to that of adrenocortical neoplasms of conventional type [3]. Recently, few case reports showed incidental adrenocortical neoplasms with either simultaneous or subsequent identification of papillary thyroid cancer [2, 7]. In conclusion, to the best of our knowledge, this is the first reported case with concomitant borderline malignant potential OAN and HCC in a four-year interval after initial resection of the adrenal mass. Moreover, none of hereditary syndromes or mutations is associated with these two described neoplasms.
